# Transcriptional Control of Dual Transporters Involved in α-Ketoglutarate Utilization Reveals Their Distinct Roles in Uropathogenic *Escherichia coli*

**DOI:** 10.3389/fmicb.2017.00275

**Published:** 2017-02-21

**Authors:** Wentong Cai, Xuwang Cai, Yongwu Yang, Shigan Yan, Haibin Zhang

**Affiliations:** ^1^Department of Veterinary Preventive Medicine, College of Veterinary Medicine, Nanjing Agricultural UniversityNanjing, China; ^2^State Key Laboratory of Agricultural Microbiology, College of Veterinary Medicine, Huazhong Agricultural UniversityWuhan, China; ^3^School of Bioengineering, Shandong Provincial Key Laboratory of Microbial Engineering, Qilu University of TechnologyJinan, China; ^4^Department of Clinical Veterinary Science, College of Veterinary Medicine, Nanjing Agricultural UniversityNanjing, China

**Keywords:** uropathogenic *Escherichia coli*, transcriptional regulation, α-ketoglutarate, transporter, sigma factor

## Abstract

Uropathogenic *Escherichia coli* (UPEC) are the primary causative agents of urinary tract infections. Some UPEC isolates are able to infect renal proximal tubule cells, and can potentially cause pyelonephritis. We have previously shown that to fulfill their physiological roles renal proximal tubule cells accumulate high concentrations of α-ketoglutarate (KG) and that gene cluster *c5032*–*c5039* contribute to anaerobic utilization of KG by UPEC str. CFT073, thereby promoting its *in vivo* fitness. Given the importance of utilizing KG for UPEC, this study is designed to investigate the roles of two transporters KgtP and C5038 in KG utilization, their transcriptional regulation, and their contributions to UPEC fitness *in vivo*. Our phylogenetic analyses support that *kgtP* is a widely conserved locus in commensal and pathogenic *E. coli*, while UPEC-associated *c5038* was acquired through horizontal gene transfer. Global anaerobic transcriptional regulators Fumarate and nitrate reduction (FNR) and ArcA induced *c5038* expression in anaerobiosis, and C5038 played a major role in anaerobic growth on KG. KgtP was required for aerobic growth on KG, and its expression was repressed by FNR and ArcA under anaerobic conditions. Analyses of FNR and ArcA binding sites and results of EMS assays suggest that FNR and ArcA likely inhibit *kgtP* expression through binding to the –35 region of *kgtP* promoter and occluding the occupancy of RNA polymerases. Gene *c5038* can be specifically induced by KG, whereas the expression of *kgtP* does not respond to KG, yet can be stimulated during growth on glycerol. In addition, *c5038* and *kgtP* expression were further shown to be controlled by different alternative sigma factors RpoN and RpoS, respectively. Furthermore, dual-strain competition assays in a murine model showed that *c5038* mutant but not *kgtP* mutant was outcompeted by the wild-type strain during the colonization of murine bladders and kidneys, highlighting the importance of C5038 under *in vivo* conditions. Therefore, different transcriptional regulation led to distinct roles played by C5038 and KgtP in KG utilization and fitness *in vivo*. This study thus potentially expanded our understanding of UPEC pathobiology.

## Introduction

Urinary tract infection (UTI) is one of the most common bacterial infections in humans, and represents a significant clinical issue worldwide (Foxman, 2010). The primary causative agent of UTIs is uropathogenic *Escherichia coli* (UPEC), which is responsible for ∼80–90% of community-acquired UTIs (Russo and Johnson, 2003; Spurbeck and Mobley, 2013). Despite that most previous studies of UPEC pathogenesis have been focused on traditional virulence factors, increasing attention is being paid to UPEC’s metabolic adaptive mechanisms, which impact colonization and survival in host’s urinary tract (Alteri and Mobley, 2015; Conover et al., 2016). UPECs have evolved multiple systems and strategies to snatch needed iron, combatting extremely low iron availability within host (Opal et al., 1990; Russo et al., 2001, 2002; Torres et al., 2001). Possession of a complete *dsdCXA* locus responsible for the detoxification of D-serine allows UPEC to use D-serine as the sole carbon and nitrogen source in urine (Roesch et al., 2003). In addition, most UPEC strains carry a genomic island involved in utilizing α-ketoglutarate (KG) under anaerobic conditions, which significantly increased UPEC’s fitness in a mouse model for UTI (Cai et al., 2013). KG is an abundant metabolite in the UPEC’s infection site – renal proximal tubule cells (Mobley et al., 1990; Chassin et al., 2008; Melican et al., 2008; Pichon et al., 2009), with an intracellular concentration of up to 400 μM (Boyd and Goldstein, 1979; Martin et al., 1989; Pritchard, 1995). KG lies at the intersection between the carbon and nitrogen metabolic pathways, and has long been recognized to coordinate carbon and nitrogen metabolism (Kim and Gadd, 2008).

A BLAST search revealed that the great majority of UPEC strains encode at least two transporters possibly associated with utilization of KG (Cai et al., 2013). A genomic island-encoded gene *c5038* was predicted to encode a putative dicarboxylate transporter with 13 transmembrane alpha-helices, showing 49% similarity to citrate/succinate antiporter CitT (Pos et al., 1998) at the amino acid level. Its expression is activated by two-component regulatory system (TCS) KguSR in response to KG under anaerobic conditions (Cai et al., 2013). Fumarate and nitrate reduction (FNR) positively regulates *c5038* expression by directly affecting the expression of KguSR (KG utilization sensor and regulator) (Barbieri et al., 2014). The other gene, *kgtP* (KG permease), encodes a KG:H^+^ symporter in *E. coli* K-12 strain (Seol and Shatkin, 1991, 1992). Alkaline phosphatase fusion study showed that KgtP contains 12 transmembrane segments (Seol and Shatkin, 1993). Genome-wide analysis of ArcA binding sites and its modulon in *E. coli* K-12 revealed that *kgtP* is negatively affected by ArcA (aerobic respiratory control), which binds to the upstream region of *kgtP in vivo* under anaerobic conditions (Liu and De Wulf, 2004; Park et al., 2013). Despite the knowledge gained of KgtP in K-12 strain and C5038, the individual roles and transcriptional regulation of C5038 and KgtP in UPEC during KG utilization remain unknown.

In this study, we describe KgtP and C5038 contribute differentially to growth on KG under aerobic and anaerobic conditions. Further analyses of their expression levels in various growth conditions and of roles played by regulators ArcA, FNR, RpoN, RpoS, and CRP (catabolite receptor protein) can account for the findings of different contributions to growth on KG and to colonization of murine urinary tracts. Overall, our results suggest that C5038 is a specialized, anaerobic KG importer, but KgtP is a generalized, aerobic KG importer. This report, therefore, should substantially improve our understanding of UPEC physiology.

## Materials and Methods

### Ethics Statement

All animal experimental procedures were conducted according to the guidelines of Experimental Animal Management Measures of Jiangsu Province and were approved by the Laboratory Animal Monitoring Committee of Jiangsu Province (China).

### Bacterial Strains and Culture Conditions

Strains and plasmids used in this study are listed in Supplementary Table S1. Aerobic growth was achieved by shaking in air at 160 rpm, and anaerobic growth by incubating in a BugBox chamber (Ruskinn, UK) filled with a gas mixture (N_2_, 85%; CO_2_, 5%; H_2_, 10%). For genetic manipulations, all *E. coli* strains were grown routinely in lysogenic broth (LB) medium (OXOID). For growth and gene expression studies, bacteria were generally grown aerobically or anaerobically in M9 minimal salts with certain carbon sources indicated, supplemented with 2 mM MgSO_4_, 0.1 mM CaCl_2_, and 1 μg ml^-1^ vitamin B1 (Cai et al., 2013). Glucose (0.4% m/v) or glycerol (0.4% v/v) (Sinopharm, China) was added as energy substrates, as indicated. When used, electron acceptor trimethylamine *N*-oxide (TMAO) and dicarboxylates were present at 40 mM. Selective antibiotics and IPTG (Isopropyl β-D-1-thiogalactopyranoside) were added when necessary at the following concentrations: ampicillin (Amp), 100 μg ml^-1^; kanamycin (Kan), 50 μg ml^-1^;chloramphenicol (Chl), 25 μg ml^-1^; IPTG, 1 mM. All reagents were purchased from Sigma-Aldrich unless otherwise noted.

For growth studies, fresh colonies of wild-type (WT) CFT073 and its derivative mutant strains were picked and inoculated in 3 ml LB medium. After OD_600_ of bacterial culture reached about 1.0, bacteria were washed once with phosphate-buffered saline (PBS) and normalized, followed by being subcultured 1:100 into M9 medium. OD_600_ value at different time points was measured and recorded using spectrophotometer (Eppendorf, basic model).

### Recombinant DNA Techniques

Polymerase chain reaction (PCR), DNA ligation, electroporation and DNA gel electrophoresis were performed according to Sambrook and Russell (2001) unless otherwise indicated. All oligonucleotide primers were purchased from BGI Technology Solutions Co., Ltd. (BGI, Guangzhou, China) and are listed in Supplementary Table S2. All restriction and DNA-modifying enzymes were purchased from New England Biolabs and were used based on the suppliers’ recommendations. Recombinant plasmids, PCR products, and restriction fragments were purified using TaKaRa PCR purification kit or gel extraction kit (TaKaRa, a Clontech company) as recommended by the supplier. DNA sequencing was performed at Shanghai Sunny Biotech Co., Ltd. DNA and amino acid sequence analyses were performed using Clone Manager software (Scientific & Educational Software, Morrisville, NC, USA) and the search engine^1^ was used to find homologous sequences. ClustalW program was used to perform multiple sequences alignments. Phylogenetic trees were made using MEGA software (Tamura et al., 2011; Hall, 2013).

Deletion mutants were constructed using the lambda red recombinase system described by Datsenko and Wanner (2000). For complementation studies, the coding sequences of genes plus their promoter regions (400 bp upstream of start codons) were amplified from the CFT073 genome and independently cloned into pGEN-MCS (Lane et al., 2007) using *Eco*RI and *Sal*I restriction sites. Plasmid pCJ112 for plasmid-borne LacZ fusion studies was constructed by replacing the R6K origin in pVIK112 [this plasmid was created for making chromosomal transcriptional fusions of *lacZ* reporter gene (Kalogeraki and Winans, 1997)] with p15A origin from pBAD30 (Guzman et al., 1995) plasmid using *Eco*RI and *Bam*HI restriction sites (See Supplementary Figure S1 for detail). The resulting plasmid was tested to show the functionality and an undetectable basal level without inserting any promoters upstream of promoterless *lacZ*. P_bla_ constitutively expressed promoter was cloned from pGEN-MCS plasmid by amplifying 400 bp upstream of *bla* gene as previous described (Sperandio et al., 1999). All constructs were verified by Sanger Sequencing (Shanghai Sunny Biotech Co., Ltd).

### β-Galactosidase Assays

Overnight LB cultures of CFT073 and its derivative strains containing the gene of interest-*lacZ* fusions were washed with PBS once and then were diluted 1:100 in LB or M9 medium with the carbon sources indicated. Empty vector pCJ112 and pCJ112 containing P_bla_-*lacZ* were used under all conditions as controls. Cultures were grown at 37°C to log phase or stationary phase. Samples were diluted 1:1 in Z buffer and assayed for β-galactosidase activity using ortho-Nitrophenyl-β-galactoside (ONPG) as a substrate as described previously (Miller, 1972).

### 5’-RACE (Rapid Amplification of cDNA Ends) PCR to Identify Transcriptional Start Site (TSS)

The transcriptional start site (TSS) site of *c5038* was identified by using ExactSTART 5′- and 3′ -RACE Kit (Epicentre) with some modifications. Briefly, bacteria were grown in M9 media supplemented with glycerol and KG to log phase. RNA was stabilized by RNAprotect Bacterial Reagent (QIAGEN) and extracted using an RNeasy Mini Kit (QIAGEN) with a 1-h in-tube DNase digestion (QIAGEN) to remove possible DNA contamination according to the manufacturer’s instructions. Complete removal of DNA contamination was confirmed by using reverse transcription PCR. Three biological replicates of each sample were prepared. The concentration of RNA was determined using a Spectrophotometer (Eppendorf, basic model). The Steps A and B were omitted as they were designed for treating Eukaryotic RNA. About 5 μg of RNA were used for the Step C in the manual: 5’-RACE Acceptor Oligo Ligation step. Control reactions were included according to the manual. The primers which yielded reliable PCR products were listed in Supplementary Table S2.

### Electrophoretic Mobility Shift Assays

To study the binding of proteins of interest to DNA probes, electrophoretic mobility shift assays (EMSAs) were performed using the commercialized EMSA kit (Invitrogen, Carlsbad, CA, USA) (Shen et al., 2011; Cai et al., 2013). pET28a prokaryotic expression system (Novagen) was used to over-produce recombinant proteins. His_6_-ArcA (Jiang et al., 2015), His_6_-(FNR-FNR)2 (Shan et al., 2012) and His_6_-RpoN fusion proteins were individually purified to homogeneity using Ni-NTA Spin Columns (QIAGEN) and dialyzed against the binding buffer. Protein concentrations were measured using BCA protein assay kit (Pierce). DNA probes (230 bp upstream of the start codon of *kgtP* or *c5038*) were PCR amplified using specific primers and gel-purified. The negative control probe was amplified from the coding region of Chloramphenicol resistance gene-*cat*. The concentrations of DNA fragments were determined by Biospectrometer (Eppendorf). EMSAs were performed by adding increasing amounts of purified fusion proteins (0240 nM) to DNA probes (10 nM) in binding buffer [10 mM Tris (pH 7.5), 1 mM EDTA, 1 mM dithiothreitol, 50 mM KCl, 50 mM MgCl_2_, 1 μg ml^-1^ bovine serum albumin (NEB)) and incubating for 30 min at room temperature (His_6_-ArcA and His_6_-RpoN), or 20 min at 37°C (His_6_-(FNR-FNR)2]. Reaction mixtures were then mixed with the loading dye (bromophenol blue, 0.25%; xylene cyanol FF, 0.25%; Ficoll 400, 15%), followed by electrophoresis on a 6% polyacrylamide gel in 0.5 × TBE buffer (44.5 mM Tris, 44.5 mM boric acid, 1 mM EDTA, pH 8.0) at 200V for 45 min in an ice-bath. The gel was stained in 0.5 × TBE buffer containing 1 × SYBR Gold nucleic acid staining solution (Life technology, USA) for 15 min. Gels were then visualized and photographed using GelDoc-it Imager (UVP).

### Experimental UTIs

Mouse infection studies were performed according to the methods of Johnson et al. (2005). Female Balb/C mice (about 6 to 7 weeks of age) were anesthetized by ketamine/xylazine/Midazolam and inoculated via transurethral catheterization with a 50 μl (∼10^9^ CFU) challenge inoculum per mouse. Overnight statically grown LB cultures of CFT073 and the mutant strains were pelleted and resuspended in sterile PBS, mixed in equal number, and adjusted to make challenge inocula. To determine the initial CFU/ml, dilutions of each inoculum were plated onto LB plates with and without chloramphenicol (*kgtP*::Chl^R^ mutant) or kanamycin (*c5038*::Kan^R^ mutant). After 48 h, the mice were sacrificed and the bladder and kidneys were aseptically removed and homogenized in tubes containing PBS using a Bioprep-24 bullet homogenizer (Allsheng, Hangzhou in China). Dilutions (10 μl in each droplet) of the homogenized tissue were then spotted onto quadruplicate LB plates with and without antibiotics to determine the bacterial load. The numbers of colonies on selective plates were subtracted from those on LB plates to obtain the number of WT bacteria. A group of 10 or 6 (for complementation studies) mice for each dual-strain challenge were used to determine alterations in fitness, and assays were performed at least twice. Competitive index is defined as the ratio of mutant to the WT isolated divided by the ratio of mutant to the WT in the inoculums. the single-gene deletion mutants

### Statistical Analyses

Statistical tests were performed using GraphPad Prism (version 5.0 for Windows, San Diego, CA, USA). For animal experiments data, Wilcoxon signed rank test was used to determine significance of competitive indices with a hypothetical median of 1 (number_WT_ = number_mutant_). For other data, unpaired Student *t*-test was used to estimate differences between samples. The threshold for statistical significance was a *P-*value < 0.05.

## Results

### C5038 and KgtP Played Differential Roles in KG Utilization under Different Oxygen Tension Conditions

Gene *c5038* is encoded on a metabolic island implicated in the anaerobic utilization of KG in UPEC (Cai et al., 2013). Bioinformatic analysis revealed that C5038 belongs to the Divalent Anion:Na^+^ Symporter (DASS) Family (Saier et al., 2006). Characterized DASS members in bacteria include succinate transporter VcINDY, citrate transporter CitT, L-tartrate/succinate transporter TtdT, C_4_-dicarboxylates uptake system DccT, YbhI, and SdcS (Markovich, 2012). C5038 on average shares 29% homology with these proteins. Sequence alignment of these DASS members (Supplementary Figure S2A) revealed that C5038 contains a SAT (serine–alanine–threonine) signature, which is similar to the relatively conserved SNT (serine–asparagine–threonine) motif involved in substrates-binding (Mancusso et al., 2012). The *kgtP* locus in K-12 is 96.4% identical to *kgtP* in UPEC str. CFT073 at DNA level, with 47 differences between them. At the amino acid level, only three differences exist between them, all of which are non-charged amino acids. KgtP shares no homology with C5038 at both DNA and amino acid levels. The promoter regions of *kgtP* in K-12 and CFT073 have an 88% sequence identity to each other (Supplementary Figure S2B).

To determine the roles of C5038 and KgtP in growth of CFT073 in various media, we compared the growth of the WT, the single-gene deletion mutants (Δ*c5038* and Δ*kgtP*, respectively), and the double-gene deletion mutant (Δ*c5038*Δ*kgtP*) in different media under aerobic conditions. We observed no significant difference in growth kinetics among these strains in LB or M9 medium supplemented with glucose (Gluc) or glycerol (Glyc) as the sole carbon source (data not shown).

**FIGURE 1:**
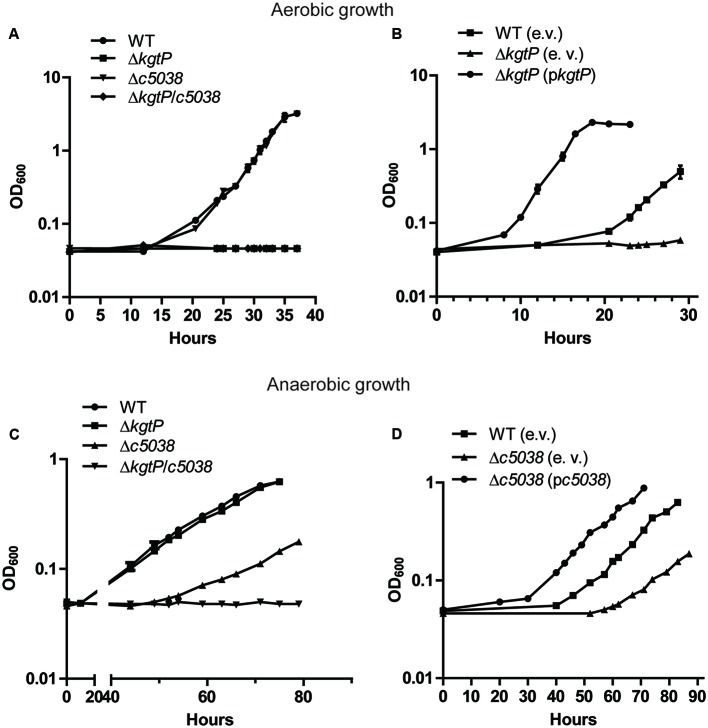
**Contributions of *c5038* and *kgtP* to growth on KG under aerobic (A,B)** and anaerobic **(C,D)** conditions. Various strains were grown in M9 medium containing KG as the sole carbon and energy source. Trimethylamine *N*-oxide (TMAO) was added as electron acceptor under anaerobic conditions. Optical density at different time points was determined. Growth curves represent the average measurement at each time point from triplicate experiments. Student *t*-test was used for statistical analysis. e. v., empty vector; p*c5038*, complementation plasmid for *c5038*; p*kgtP*, complementation plasmid for *kgtP*.

When cultured in M9 using KG as the sole carbon source under aerobic conditions, the Δ*c5038* mutant showed similar growth kinetics as WT, indicating that *c5038* was not important for growth under these conditions (**Figure 1A**). In contrast, deletion of *kgtP* abolished CFT073’s growth (*P* < 0.0001) and *kgtP* mutant displayed no detectable growth within 40 h. To rule out the possible masking effect of *kgtP* on *c5038*’s role, we compared the growth of two mutants, Δ*kgtP* mutant and Δ*c5038*Δ*kgtP* mutant. The results showed no significant difference in growth between Δ*kgtP* mutant and Δ*c5038*Δ*kgtP* mutant (**Figure 1A**), further supporting that *c5038* played a negligible role in KG utilization in aerobiosis. Additionally, the introduction of a complementation plasmid (p*kgtP*) carrying *kgtP* controlled by its native promoter restored the growth of *kgtP* mutant (**Figure 1B**). These results suggest that *kgtP* is required whereas *c5038* is dispensable for growth on KG under aerobic conditions.

Under anaerobic conditions, deletion of *kgtP* did not affect CFT073’s growth, whereas loss of *c5038* dramatically reduced its growth in M9 with KG as the sole carbon source, as compared to WT (**Figure 1C**). Transformation of the *c5038* mutant with a complementation plasmid (p*c5038*) carrying *c5038* controlled by its native promoter significantly improved *c5038* mutant’s growth (**Figure 1D**). To rule out the possible masking effect of *c5038* on *kgtP*’s role under these conditions, we compared the growth of Δ*c5038* mutant and Δ*c5038*Δ*kgtP* mutant. We observed a difference in growth between Δ*c5038* mutant and Δ*c5038*Δ*kgtP* mutant at 60 h time point and thereafter (*P* < 0.05) (**Figure 1C**). These data indicate that under anaerobic conditions, *c5038* played a major role and *kgtP* a minor role in growth on KG. Of note, in both aerobiosis and anaerobiosis, the complementation strains apparently had much shorter lag phases and higher growth rates than the WT strain carrying the empty vector. This was likely due to the fact that the complementation plasmid vector is a multi-copy plasmid (∼15 copies per cell), which can cause overexpression of *kgtP* or *c5038*.

### Anaerobiosis Induced *c5038* but Repressed *kgtP* Expression

**FIGURE 2:**
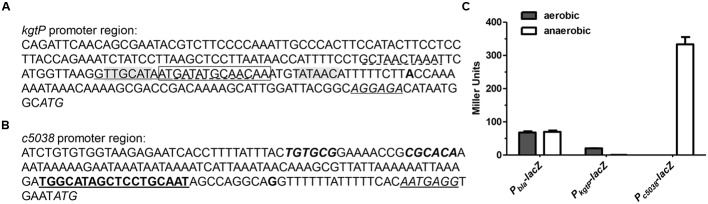
***c5038* transcription was induced whereas *kgtP* repressed in anaerobiosis. (A)** Promoter region of *kgtP*. **(B)** Promoter region of *c5038.* Italic, start codon; italic and underlined, putative ribosome binding site; bold, transcription start site; bold and underlined, RpoN binding site/σ^54^ motif (–12 and –24 regions); italic and bold, inverted repeats for KguR binding; shaded, –10 and –35 regions; Underlined (solid), perfect ArcA binding site; underlined (dashed), imperfect ArcA binding site; boxed, FNR binding site. **(C)** *kgtP* and *c5038* expression under aerobic and anaerobic conditions. Bacteria were grown aerobically or anaerobically in M9 medium containing KG as the sole carbon source. TMAO was used as an electron acceptor for anaerobic tests. Expression of *c5038*-*lacZ* or *kgtP-lacZ* was indicated by β-galactosidase activity. P_bla_-*lacZ* constitutive expression was used as a control. Bars represent means ± SEM from three independent experiments performed in triplicate.

Given that *kgtP* and *c5038* contributed differentially to growth on KG under aerobic and anaerobic conditions, we sought to unravel their transcriptional levels under these conditions. The TSS of *c5038* was obtained using 5′-RACE PCR (**Figure 2B**). The –10 and –35 sites of *kgtP* promoter were predicted by Virtual Footprint program (Munch et al., 2005) and Bprom program (Solovyev and Salamov, 2011), which presented consistent output with high reliability. The putative ribosome binding sites were also indicated. The predicted TSS of *kgtP* in CFT073 was highly similar to that in K-12 identified by 5′-RACE and deep sequencing (Cho et al., 2009). The promoter regions of *kgtP* and *c5038* were then cloned to drive promoterless *lacZ* gene on a plasmid, respectively, and the resulting plasmid constructs were individually introduced into LMP10 strain (CFT073 Δ*lacZYA*). In parallel, a control plasmid carrying constitutively expressed promoter P_bla_-*lacZ* fusion was also transformed into LMP10 strain. β-galactosidase activities were measured to indicate expression levels. As shown in **Figure 2C**, *c5038* expression was not detectable in aerobiosis, but was induced in anaerobiosis. The expression of *kgtP* was moderate under aerobic conditions, but was greatly repressed under anaerobic conditions, with a 40-fold reduction. It is also evident that anaerobic expression level of *c5038* was significantly higher than aerobic expression level of *kgtP* (about 10-fold difference). In the control strain, P_bla_-*lacZ* expression did not exhibit difference between under aerobic and anaerobic conditions. These results clearly showed that under anaerobic conditions, *c5038* was highly expressed whereas *kgtP* was severely repressed, thereby at least partially explaining the phenotypic difference in growth.

### FNR and ArcA Induced *c5038* But Repressed *kgtP* Expression

**FIGURE 3:**
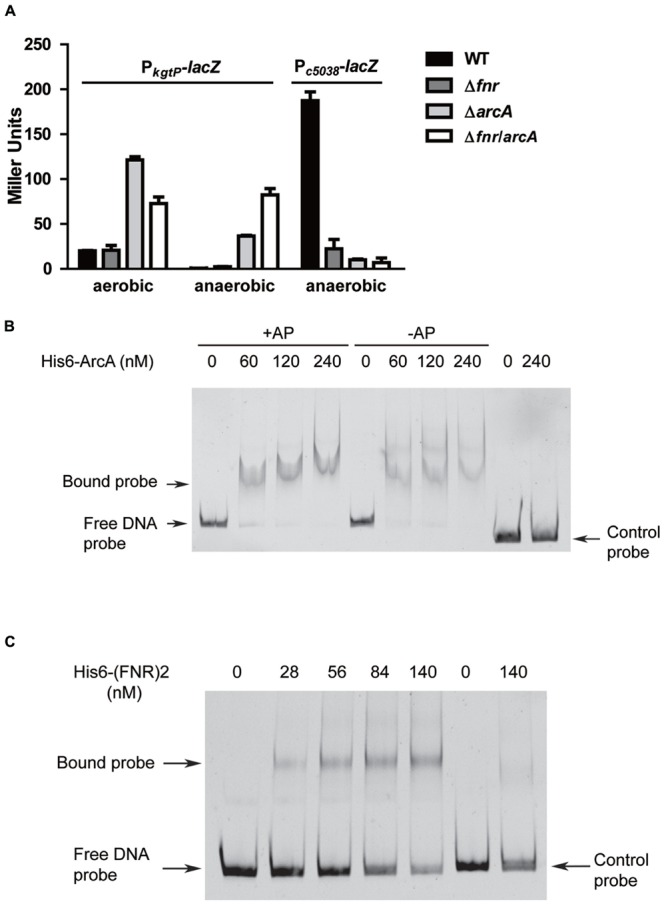
**Roles of *fnr* and *arcA* in regulating *kgtP* and *c5038* expression in response to varying oxygen tension. (A)** Loss of *fnr* or *arcA* leads to different effects on *kgtP* and *c5038* expression. Various strains were grown aerobically or anaerobically in M9 medium containing KG as the sole carbon source. TMAO was used as electron acceptor anaerobically. Expression of *c5038*-*lacZ* or *kgtP-lacZ* in different strains was represented by β-galactosidase activity. **(B)** Non-radioactive EMSA studying the binding of ArcA to *kgtP* promoter regions. **(C)** Non-radioactive EMSA studying the binding of FNR to *kgtP* promoter regions. Gel-extracted PCR products of *kgtP* promoter region and *cat* (Chl^R^ gene) coding region were used as probes. Purified His_6_-ArcA or His_6_-(FNR)2 fusion protein was added in different concentrations in each reaction mixture as indicated. DNA fragments were stained with SYBR green. AP, acetyl phosphate.

Fumarate and nitrate reduction and ArcA are two master regulators mediating bacterial adaptation to environmental oxygen availability (Constantinidou et al., 2006; Park et al., 2013; Jiang et al., 2015). To probe the regulatory roles of *arcA* and *fnr* in expression of *c5038* and *kgtP*, we examined the effects of single deletion and double deletion of *fnr* and *arcA* on expression levels of *c5038* and *kgtP* under aerobic and anaerobic conditions. Under aerobic conditions, deletion of *fnr* did not affect *kgtP* expression while loss of *arcA* upregulated *kgtP* expression about fivefold. Under anaerobic conditions, deletion of either *fnr* or *arcA* caused upregulation of *kgtP* expression (4-fold and 700-fold increase, respectively). Deletion of both *fnr* and *arcA* led to a greater increase in *kgtP* expression than individual deletions of *fnr* and *arcA*. These results indicate that both *fnr* and *arcA* negatively modulate *kgtP* expression. Further, in LMP10 strain, anaerobiosis repressed *kgtP* expression about 40-fold, as compared to aerobic expression, whereas in LMP10Δ*fnr* and LMP10Δ*arcA* mutants, anaerobiosis repressed *kgtP* expression 10-fold and 4-fold, respectively (**Figure 3A**). These results clearly demonstrate that in either *fnr* or *arcA* mutant, anaerobic repression of *kgtP* was partially relieved compared with that in their parental strain LMP10, suggesting both *fnr* and *arcA* are involved in the anaerobic repression, with ArcA being a major player.

We next tried to understand the mechanisms by which ArcA and FNR repress the expression of *kgtP*. Sequence analyses of promoters repressed by ArcA or FNR suggest that ArcA and FNR inhibit gene expression through competitive binding to –10 or/and –35 region, thereby blocking occupancy of RNA polymerases (Myers et al., 2013; Park et al., 2013). Using a recently developed binding consensus sequence of ArcA ([GT][TA][TA][AG][AC][AT][AT][AT][AT][AT], each pair of letters in each bracket indicate two most frequent bases in that position, with the first being the most frequent) (Park et al., 2013), we were able to identify one perfect match (GTTGCATAAT) that overlaps the putative –35 region and two imperfect matches close to the perfect one (**Figure 2A**). Similarly, using a recently developed binding consensus sequence of FNR ([TA][TC][GA][AC][TC]nnnn[AG][TA][CT][AG][AC]) (Myers et al., 2013), we were able to identify a perfect match (ATGATatgcAACAA) that lies between –10 and –35 region. To test whether ArcA and FNR directly binds to the promoter region of *kgtP*, EMS assays were carried out. DNA fragments with sizes around 230 bp containing the predicted sites were prepared. Purified His_6_-tagged ArcA and FNR variant (FNRD154A) proteins (Shan et al., 2012) were obtained by nickel affinity chromatography. As shown in **Figure 3B**, the DNA probe of *kgtP* promoter region can be shifted by ArcA protein, but the negative control probe cannot be shifted. With the same amounts of protein, the presence of acetyl phosphate appears to not affect binding of ArcA to DNA under our testing conditions. Also, FNR recombinant protein can shift the DNA probe of *kgtP*, but not the control probe (**Figure 3C**). These results indicate that ArcA and FNR can bind to the promoter region of *kgtP* directly. Altogether, these data suggest that ArcA and FNR likely repress *kgtP* expression through binding to promoter region and occluding the occupancy of RNAP.

For *c5038*, deletion of either *fnr* or *arcA* or both resulted in a significant reduction in *c5038*-*lacZ* expression in comparison to that in the LMP10 strain (*P* < 0.01), suggesting both *fnr* and *arcA* positively modulate *c5038* expression. We next used EMSA to test if ArcA directly associates with DNA probe of *c5038* promoter region. The results show that ArcA protein cannot shift the DNA probes (Supplementary Figure S3), raising the possibility that ArcA may not regulate *c5038* expression directly. Taken together, these data indicate that FNR and ArcA stimulated *c5038* but repressed *kgtP* expression.

### Response of *c5038* and *kgtP* Expression to Different Stimuli and the Role of CRP

**FIGURE 4:**
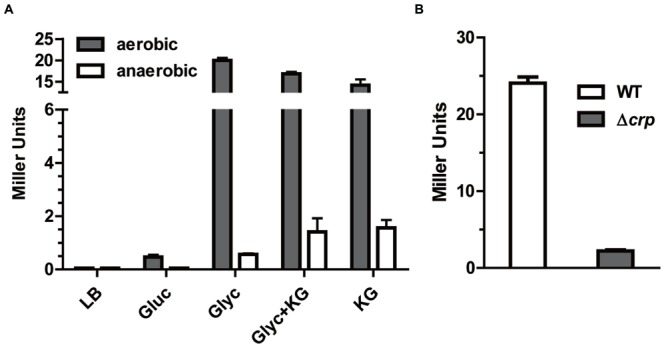
**Response of *kgtP* expression to growth in different media and contribution of global regulator CRP. (A)** *kgtP* expression does not respond to the presence of KG. Bacteria were grown aerobically or anaerobically in different media containing the indicated carbon and energy source. TMAO was used as an electron acceptor for anaerobic tests. Gluc, glucose; Glyc, glycerol. **(B)** Role of CRP in *kgtP* expression. Bacteria were grown in M9 containing glycerol as sole carbon source. Expression of *kgtP-lacZ* was indicated by β-galactosidase activity. Bars represent means ± SEM from three independent experiments performed in triplicate.

In contrast to *c5038*, what stimuli induce *kgtP*’s expression is still unclear. To test if the expression of *kgtP* is influenced by the addition of KG, we measured and compared the expression levels of *kgtP* during bacterial growth in M9 (Glyc), M9 (Glyc and KG), and M9 (KG), respectively. Surprisingly, *kgtP* expression in all three media is comparable (**Figure 4A**), indicating that *kgtP* expression does not respond to the presence of KG. We next tested and compared the expression of *kgtP* in LB rich medium, M9 (Gluc), and M9 (Glyc). The results showed that the order of *kgtP* expression levels is as follows: M9 (Glyc) > M9 (Gluc) > LB.

The cAMP-CRP complex is a global regulatory element that induces a number of genes involved in the utilization of carbons in the absence of glucose (Botsford and Harman, 1992; Kolb et al., 1993), including glycerol catabolic regulon (Iuchi et al., 1990). We therefore examined whether CRP contributes to the regulation of *kgtP* expression. As shown in **Figure 4B**, *kgtP* expression in the strain lacking *crp* was downregulated, as compared to the parental strain LMP10. This result indicates that CRP positively regulates *kgtP* expression. Collectively, these data suggest that *kgtP* might be responsive to the carbon state of the cell, and be used for carbon scavenging.

### Roles of Different Alternative Sigma Factors in the Expression of *c5038* and *kgtP*

**FIGURE 5:**
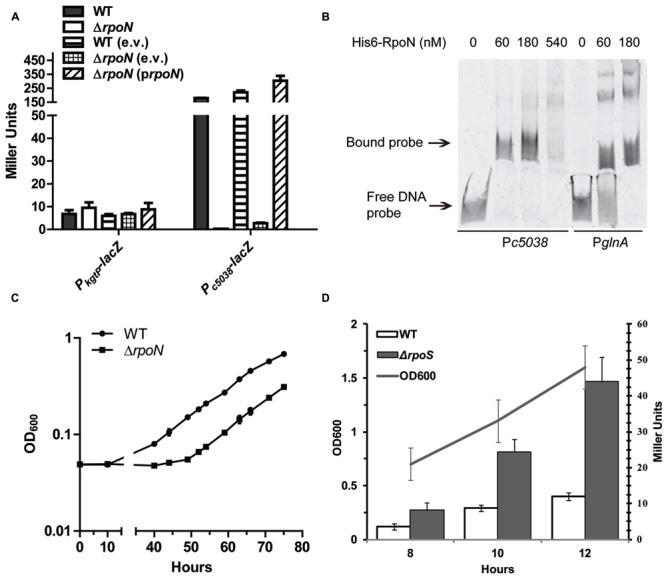
**Role of alternative σ^54^ encoded by *rpoN* in *kgtP* and *c5038* expression. (A)** Loss of RpoN affects *kgtP* and *c5038* expression differently. Various strains were grown aerobically (*kgtP*) or anaerobically (*c5038*) in M9 medium containing glycerol (*kgtP*) or KG (*c5038*) as the sole carbon source. TMAO was used as electron acceptor anaerobically. Expression of *c5038*-*lacZ* or *kgtP-lacZ* in different strains was represented by the β-galactosidase activity. e.v., empty vector; p*rpoN*, complementation plasmid carrying *rpoN*. **(B)** Non-radioactive EMSA studying the binding of RpoN to promoter regions. PCR products of *glnA* (right three lanes) and *c5038* (left four lanes) promoter regions were used as probes. Purified His_6_-RpoN fusion protein was added in different concentration in each reaction mixture as indicated. DNA fragments were stained with SYBR green. **(C)** Growth of the wild-type (WT) and *rpoN* deletion mutant. Bacteria were cultured in M9 minimal medium supplemented with KG as the sole carbon source and TMAO as an electron acceptor. Samples were taken at the indicated time points, and their OD_600_ was measured. Each point in the growth curve represents the average measurement from triplicate experiments. **(D)** The role of *rpoS* in *kgtP* expression in different growth phases. Bacteria were grown aerobically in M9 medium containing glycerol as the sole carbon source. Expression of *kgtP-lacZ* was represented by the β-galactosidase activity.

KguR involved in direct activation of *c5038* was predicted to belong to the category of bacterial enhancer-binding proteins (bEBPs) which interact with σ^54^ factor (Bush and Dixon, 2012), raising the possibility that *c5038* is a new member of the σ^54^ regulon. The “A,” –30 site relative to the start codon was identified to be the TSS of *c5038* (**Figure 2B**). Further analysis of the promoter region of *c5038* did reveal a motif that is highly similar to the σ^54^ binding consensus “–24 to –12” region (**Figure 2B**), suggesting that *c5038* transcription might depend on sigma54. To assess the contribution of *rpoN* (the gene coding for sigma54) in *c5038* expression, we compared the expression levels of *c5038* in LMP10 with that in LMP10Δ*rpoN*. As shown in **Figure 5A**, deletion of *rpoN* abolished the KG induction of *c5038* expression, as compared to parental strain LMP10. To ensure that no unwanted mutation was responsible for such phenotype, a complementation study was performed by introducing a copy of plasmid-borne *rpoN* controlled by its putative native promoter. The result showed that with *rpoN* reintroduced, the expression level of *c5038* was rescued.

Sigma54 protein was shown to be capable of binding to target promoter regions independent of core RNA polymerase (Shingler, 2011). To evaluate if σ^54^ protein directly associates with *c5038* promoter region, EMSA was used to study the binding of σ^54^ proteins to DNA molecules. σ^54^ was over-produced in *E. coli* as His_6_-RpoN fusion protein, which was then purified to homogeneity against nickel column. *glnA* promoter region, which was previously shown to be directly associated with RpoN (Huo et al., 2006), was included as a positive control; on the other hand, *xylA* promoter region, a member of σ^70^ regulon (Song and Park, 1997; Desai and Rao, 2010), was included as a negative control. As we expected, His_6_-RpoN fusion protein can shift P*_glnA_* and P*_c5038_* DNA probes, but not the P*_xylA_* (**Figure 5B**; Supplementary Figure S4) probe. Altogether, these data indicate that *c5038* is a new member in the RpoN regulon.

Because RpoN is required for *c5038* expression which is important for growth of CFT073 on KG, we then examined the growth kinetics of the WT and Δ*rpoN* mutant in M9 (KG) containing abundant nitrogen source NH_4_Cl under anaerobic conditions. The results demonstrate that Δ*rpoN* mutants had a longer lag phase and grew significantly slower than their parental WT strain (*P* < 0.001) (**Figure 5C**). These data suggest that RpoN contributes to anaerobic utilization of KG, possibly through regulating *c5038* expression.

In contrast to *c5038*, deletion of *rpoN* had no effect on *kgtP* expression (**Figure 5A**). We have shown earlier that *kgtP* expression remains high in M9 (Glyc), which is considered to be a carbon- and amino acid-limited environment. RpoS, also known as sigma38 factor, is a stress-responsive global regulator, which is induced under stress conditions such as carbon, phosphorus, nitrogen, or amino acid scarcity (Notley and Ferenci, 1996). Thus, *rpoS* was deleted from LMP10 strain, and its effects on *kgtP* expression were determined. Unexpectedly, loss of RpoS increased *kgtP* expression by about twofold, irrespective of growth phase (**Figure 5D**). Therefore, RpoS is a negative modulator of *kgtP* expression.

### *c5038* But Not *kgtP* Contributed to UPEC Fitness *In vivo*

**FIGURE 6:**
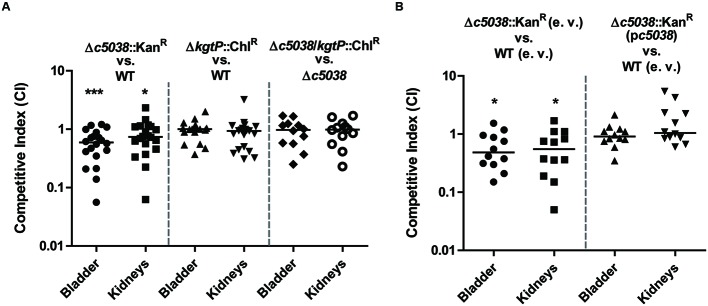
**Fitness of *c5038* and *kgtP* mutants during co-infections with the WT.** Bacteria were recovered from mice transurethrally challenged with a 1:1 mixture of two strains. Colonies on LB plates and LB plates containing the indicated antibiotics were enumerated. Competitive indices at 48 hpi were determined by dividing the ratio of two strains recovered by the ratio of the same two strains in the inoculum. Each group contains 10 or 6 mice, and experiments were performed at least twice. Bars represent the medians, and a Wilcoxon signed rank test was used to determine the significance of CI against a hypothetical median of 1. The threshold for statistical significance was a *P-*value < 0.05. ^∗^*P* < 0.05; ^∗∗∗^*P* < 0.001. **(A)** *In vivo* competition of Δ*c5038*::Kan^R^/WT, Δ*kgtP*::Chl^R^/WT, and Δ*c5038*/*kgtP*::Chl^R^/Δ*c5038*. **(B)** *In vivo* complementation study of *c5038*.

Now that *c5038* and *kgtP* were demonstrated to play differential roles *in vitro*, we next tried to investigate if loss of *c5038* or *kgtP* affects UPEC fitness *in vivo*. Mice were challenged transurethrally with a 1:1 mixture of WT and a mutant, followed by recovering bacteria from tissues 48 h post inoculation and calculating competitive indices. Note that when inoculated in LB medium with 1:1 ratio, any two strains contained in the inoculums were recovered equally in numbers during exponential growth phase, indicating that they were equally fit *in vitro*. **Figure 6A** showed that the *c5038* mutant was significantly outcompeted by the WT in both bladder and kidneys (*P*-value equals 0.0005 and 0.0458, respectively). *In vivo* complementation experiments were performed to confirm that the mutation is not polar and the disadvantage is not caused by a secondary mutation (**Figure 6B**). The stable low-copy pGEN plasmid was used as it was shown to be well maintained in CFT073 up to 48 h even in the absence of antibiotic pressure. The Δ*c5038* mutant carrying empty vector displayed the expected competitive disadvantage in bladder and kidneys when co-challenging mice with the WT containing pGEN (*P*-value equals 0.0161 and 0.0342, respectively) whereas Δ*c5038* harboring a copy of *c5038* on pGEN plasmid was equally fit as WT (pGEN). Therefore, reintroduction the *c5038* gene into the *c5038* mutant can potentially complement the negative effect on colonization caused by mutation of *c5038*.

In contrast to the *c5038* mutant, the *kgtP* mutant colonized murine urinary tracts as well as the WT (**Figure 6A**, CI was not significantly different from the hypothetical value 1). To rule out the possible masking effect of *c5038* on *kgtP*, we tested the effects of mutating *kgtP* in Δ*c5038* genetic background on fitness *in vivo*. Deletion of *kgtP* in Δ*c5038* background had no impact on colonization of mice by CFT073, further corroborating that *kgtP* is not important for colonization of mice by UPEC. Taken together, these results suggest that *c5038* but not *kgtP* is important for UPEC fitness *in vivo*.

### Phylogenetic Analysis of *c5038* and *kgtP*

In light of distinct roles of C5038 and KgtP in KG utilization and *in vivo* fitness of CFT073, their phylogenetic trees were constructed in order to understand their evolutionary routes (*Escherichia* spp. strains were excluded). Comparing the two phylogenetic trees (Supplementary Figures S5A,B), it is evident that the two transporters have different patterns: KgtP is clustered with many *E. coli* close relatives whereas C5038 is clustered with only two of them, with others being Pseudomonads and Burkholderiales. These data strongly indicate that C5038 and KgtP have distinct evolutionary trajectories, supporting our suggestion that *c5038* was acquired through horizontal gene transfer.

## Discussion

Bacterial pathogens usually possess more than one system/factor to execute certain cellular function critical for pathogenicity (Brussow et al., 2004). In UPEC, multiple fimbrial genes and iron acquisition systems exist and contribute to UPEC’s fitness in different host niches and environments (Garcia et al., 2011; Spurbeck et al., 2011). In this study, we described multiple aspects of transcriptional regulation of two transporters, C5038 and KgtP, and effects of their regulation on physiological roles played by the two transporters. The expression of *c5038* was induced solely by KG under anaerobic conditions, which was mediated by ArcA and FNR regulators responsible for adaptation to low-oxygen environments. Thus, it can be expected that possession of *c5038* can significantly increase UPEC’s growth on KG anaerobically (**Figures 1C,D**). Phylogenetic analysis supports that *c5038* was acquired through horizontal gene transfer. Further, *c5038* is highly prevalent in UPEC strains (78%), but very lowly in diarrheagenic *E. coli* strains (0%) (Cai et al., 2013). These findings prompted us to reason that C5038 is a specialized protein that carries out important functions for UPEC under *in vivo* conditions. During infection by UPEC, host sites such as renal proximal tubule cells could provide abundant KG (Martin et al., 1989; Pritchard, 1995) while oxygen tension is low (Melican et al., 2008). Indeed, loss of *c5038* led to reduced fitness during colonization of murine urinary tracts by UPEC (**Figure 6A**). Although the *c5038* mutant was outcompeted less than twofold, it is plausible that under our testing conditions, C5038 did not exert effects to its full potential. Acquisition of genes or factors that promote fitness of bacterial pathogens is a recurring theme. For example, in response to tetrathionate, genomic island-encoded loci *ttrABC* provide a growth advantage for *Salmonella* by utilizing tetrathionate anaerobically as an electron receptor produced by inflamed guts (Price-Carter et al., 2001; Winter et al., 2010).

The expression of *kgtP* is not inducible by KG and appears to be constitutively expressed in K-12 strain, implying that *kgtP* is expressed at similar levels in different environments (Seol and Shatkin, 1992). We did show here that *kgtP* expression in CFT073 does not respond to the addition of KG, but its expression was high in M9 (Glyc) and low in M9 (Gluc) and LB. This was consistent with previous KG accumulation assays demonstrating that maximal intracellular concentration of KG was achieved for bacteria grown on glycerol, followed by those on Gluc and LB (Seol and Shatkin, 1992). Because one of the major stresses *E. coli* face during growth on glycerol is limited carbon availability, it is tempting to hypothesize that *kgtP* senses carbon availability of bacterial cells and that importing KG could scavenge carbons (Yan et al., 2011) and help improve carbon status, considering the role of KG in providing carbon skeletons for both TCA cycle (tricarboxylic acid cycle) and amino acid metabolism. Supporting data is that the expression level of *kgtP* was higher in stationary phase when carbon sources are mostly depleted (Supplementary Figure S6), and was regulated by CRP and stress sigma factor σ^S^. However, more biochemical evidence is needed to further address that hypothesis. Similar to KgtP in K-12, KgtP in UPEC str. CFT073 is also required for aerobic growth using KG as the sole carbon source. On the contrary, KgtP only played a minor role in bacterial growth on KG under anaerobic conditions (**Figure 1C**). Expectedly, loss of KgtP did not result in any competitive disadvantage in the animal model. Overall, we for the first time revealed *kgtP* is subject to regulation by oxygen tension, which was mediated by ArcA and FNR.

The TCA cycle is a bi-functional pathway that generates ATP during the catabolic process and supplies skeletons in the anabolic process of biosynthesis (Kim and Gadd, 2008). As a result, transport, assimilation, and conversion of intermediates in TCA cycle are subject to regulation in response to oxygen availability (Nakano et al., 1998; Kim and Gadd, 2008). For four-carbon (C_4_)-dicarboxylates in TCA cycle including succinate, fumarate, and malate, *E. coli* possess both aerobic and anaerobic transporters, namely, DctA (Davies et al., 1999) and DcuABC (Zientz et al., 1996; Golby et al., 1998). Similar to DctA, KgtP requires protons to import its substrate-KG (Seol and Shatkin, 1992). Similar to DcuABC which exchanges their substrates with succinate, we also found that overexpression of *c5038* led to decreased succinate assimilation, suggesting that C5038 likely exported succinate (data not shown). To ensure optimal assimilation of KG, KgtP as a proton symporter needs to be inhibited in low-oxygen environments in order to save energy, whereas C5038 can function in a substrate-product exchange fashion which is less energy-consuming and thus highly desirable. Indeed, our data show that FNR and ArcA co-repress *kgtP* but co-activate *c5038* expression, emphasizing roles of FNR and ArcA in promoting a coordinated response to KG in anaerobic environments. Likewise, for C_4_-dicarboxylate transporters, DctA is repressed by ArcA under anaerobic conditions (Davies et al., 1999) while Dcu system is activated by FNR under anaerobic conditions (Engel et al., 1992). Conceivably, possessing functional alternatives with optimized regulatory patterns for importing KG should greatly promote UPEC’s fitness in changing environments. To our knowledge, for the first time, we reveal bacteria carry both aerobic and anaerobic transporter systems for KG. The conformity between C_4_- and C_5_-dicarboxylates transporters makes us speculate that such transport systems and regulatory patterns might be widely existed for other important metabolites in bacteria.

Since FNR activates *arcA* expression under anaerobic conditions (Compan and Touati, 1994), it raises a question of whether FNR can affect *kgtP* expression through ArcA. Our data showed that in the absence of *arcA*, mutation of *fnr* can still increase the expression of *kgtP* by about twofold, indicating that FNR can regulate *kgtP* independent of ArcA. ArcA represses *kgtP* expression to a higher degree than FNR does, this can be at least partially attributable to the fact that ArcA can potentially bind to three putative binding sites, whereas FNR can only bind to one site. By binding to more than one site, ArcA has a potential to form multimer (Pellicer et al., 1999; Jeon et al., 2001); in contrast, FNR usually works as a dimer (Lazazzera et al., 1996; Jervis and Green, 2007). In the *arcA* mutant, *kgtP* expression in aerobiosis was higher than that in anaerobiosis, this may be due to that (1) *fnr* can still repress gene expression in anaerobiosis when *arcA* is absent, (2) there are other activating elements (probably cAMP-CRP) that function better under aerobic conditions, or (3) the metabolic changes caused by *arcA* deletion.

Like TCS response regulator KguR and inducer KG, sigma54 factor is also essential in activating *c5038* expression, thereby adding one more level of control in the existing regulatory network for orchestrating *c*5038 expression. It is well established that members of sigma54 regulon are primarily involved in nitrogen metabolism, although exceptions exist (Shingler, 2011). The inclusion of sigma54 in *c*5038 regulatory network is expected to have great biological significance as nitrogen availability and KG accumulation are inversely correlated (Reyes-Ramirez et al., 2001; Yan et al., 2011). Recent studies have suggested that UPEC can encounter environments with limited nitrogen source during infection (Snyder et al., 2004). Therefore, it is likely that expression of *c*5038 helps accumulate KG and further to adapt to nitrogen-limited urinary tracts (Snyder et al., 2004; Hagan and Mobley, 2007). Notably, RpoN coding for sigma54 factor and a bEBP regulator MifR were both required for the expression of KG transporter PA5530 (Lundgren et al., 2014) in *Pseudomonas aeruginosa*, highlighting the possibility of commonly using a similar mechanism to link KG and nitrogen metabolism. By contrast, sigma54 factor does not contribute to *kgtP* expression, further distinguishing it from *c*5038.

In summary, we employed bioinformatic, biochemical, and genetic tools to study two transporters involved in KG utilization, demonstrating that C5038 is likely an anaerobic KG importer but KgtP is an aerobic KG importer. Their distinct roles can be at least in part attributed to distinctive transcriptional regulatory patterns. The development of these two systems should provide adaptive advantages for UPEC in various environments.

## Author Contributions

WC, SY, and HZ conceived and designed the experiments; WC, YY, and XC performed the experiments; WC and HZ analyzed the data; WC, SY, and HZ contributed reagents/materials/analysis tools; WC, SY, and HZ wrote the paper. All authors contributed, read and approved the final manuscript.

## Conflict of Interest Statement

The authors declare that the research was conducted in the absence of any commercial or financial relationships that could be construed as a potential conflict of interest.

## Footnotes

1 http://blast.ncbi.nlm.nih.gov/Blast.cgi
