# Supplementary material for: Transcriptional Control of Dual Transporters Involved in α-Ketoglutarate Utilization Reveals Their Distinct Roles in Uropathogenic Escherichia coli
Source: Front Microbiol. 2017 Feb 21;8:275. doi: 10.3389/fmicb.2017.00275 (PMC5318444; doi:10.3389/fmicb.2017.00275)
Supplement: Supplementary file 1 [file Data_Sheet_1.pdf]

Transcriptional control of dual transporters involved in α-ketoglutarate utilization reveals their distinct roles in uropathogenic *Escherichia coli*

Wentong Cai[a], Xuwang Cai[b], Yongwu Yang[a], Shigan Yan[c*], Haibin Zhang[a, d*]

Table S1. Bacterial strains and plasmids used in this study.

Bacterial strains and plasmids	Genotype or relevant characteristics	Source or Reference
Bacterial strains		
E. coli **DH5α**	**Plasmid propagation strain**	**Invitrogen**
**UPEC CFT073**	**Blood isolate from a patient with acute pyelonephritis**	70
**LMP10**	**UPEC CFT073 derivative, Δ*lacZYA***	22
**Δ*c5038***	**UPEC CFT073 *c5038* deletion mutant**	**This study**
**Δ*c5038* (p*c5038)***	**Δ*c5038* mutant carrying a complementation plasmid for *c5038***	**This study**
**Δ*kgtP***	**UPEC CFT073 *kgtP* deletion mutant**	**This study**
**Δ*kgtP* (p*kgtP)***	**Δ*kgtP* mutant carrying a complementation plasmid for *kgtP***	**This study**
**Δ*kgtP*Δ*c5038***	**UPEC CFT073 *kgtP*/*c5038* double deletion mutant**	**This study**
**Δ*rpoN***	**LMP10 *rpoN* deletion mutant**	**This study**
**Δ*crp***	**LMP10 *crp* deletion mutant**	**This study**
**ΔrpoS**	**LMP10 *rpoS* deletion mutant**	**This study**
Plasmids		
**pKD3**	**template for λ-Red Chl^r cassette**	71
**pKD4**	**template for λ-Red Kan^r cassette**	71
**pCP20**	**encodes FLP recombinase for removal of resistance cassette**	71
**pKD46**	**λ-Red recombinase expression**	71
**pMAL-MCS**	***malE* in PMAL-c2x was replaced by multiple cloning sites from pEGFP plasmid**	22
**pMAL-MBP/c5040**	**pMAL-c2x carrying MBP-C5040-6 ×His-tag under the control of Ptac**	22
**pBAD**	**p15A replication origin plasmid**	63
**pVIK112**	**suicide plasmid for chromosomal *lacZ* transcriptional fusion, R6K origin**	62
**pCJ112**	**R6K origin replaced by p15A origin from pBAD**	**This study**
**pGEN-MCS**	**low copy plasmid for complementation**	72
**p*rpoN***	**complementation plasmid carrying *rpoN* coding region and its predicted promoter region**	**This study**
**p*c5038***	**pGEN-MCS carrying *c5038* coding region led by its native promoter**	**This study**
**p*kgtP***	**pGEN-MCS carrying *kgtP* coding region led by its native promoter**	**This study**
**pET28**	**T7 promoter driven protein overexpression in *E. coli***	**Novagen**

**pET28-*rpoN***	**His$_6$-RpoN overexpression driven by T7 promoter**	**This study**

Table S2. Oligonucleotides used in this study.

Primers	Sequence (5'-3')
pGEN-c5038-F	AGCTGAATTCTGATGCGCTGCGTTTATTCG
pGEN-c5038-R	ACTGGTCGACTCGGCATCACAGCCATTAAG
pGEN-kgtP-F	agtcGAATTCCAGAAGTGAAACGCCGTAGC
pGEN-kgtP-R	agtcGTCGACTGGGATATCGCCGGTGCAAG
pCJ112seqF	AGCGAGTCAGTGAGCGAGGAAG
p15A-F-EcoRI	ATGCGAATTCGCATGCTGGTACCGGGCGCGGCCGCGGGCCCCACATGGAAGCCATCACAGAC
p15A-R-BamHI	GCATGGATCCTCCTCTACGCCGGACGCATC
C5038-RT-Primer	CGGCAGGATGAAGCCAAAAC
C5038-GSP2	CAATGCGTTTATCCAGCCCG
C5038-GSP5	ATCCATCGCTTCGCTGATCC
PkgtP-F	CTAC*GAATTC*ATTTGCCTGGCGGCCTTAGC
PkgtP-R	GTAC*TCTAGA*CTCCTGCCGTAATCCAATGC
Pc5038-F	AGTCGAATTCTGGTGGTAATGCGGAAGAAC
Pc5038-R	ATCGTCTAGATATCGCCCAGTGGCAGAAGG
del-fnr-F	GATCAATAAATCAGAAAAATTTAATGATATGACAGAAGGATAGTGAGTTATGCGGAAGAAgtgtaggctggagctgcttcga
del-fnr-R	ATCTAATATCGGAATTCTCTGCTGTTAAGGTTTGCTTAGACTTACTTGCTCCCTAAAAAGcatatgaatatcctccttag
del-arcA-F	AAAAGCGCCGTTTTTATTGACGGTGGTAAAGCCGATTAATCTTCCAGATCgtgtaggctggagctgcttcga
del-arcA-R	GGACTTTTGTACTTCCTGTTTCGATTTAGTTGGCAATTTAGGTAGCAAACcatatgaatatcctccttag
crp-del-F	AGCGGCGTTATCTGGCTCTGGAGAAAGCTTATAACAGAGGATAACCGCGCgtgtaggctggagctgcttcga
crp-del-R	CGGGGGAAACAAAATGGCGCGCTACCAGGTAACGCGCCACTCTGACGGGAcatatgaatatcctccttag
rpoS-del-F	TTACTCGCGGAACAGCGCTTCGATATTCAGCCCCTGCGTTTGCAGGATTTCGCGCgtgtaggctggagctgcttcga
rpoS-del-R	ATGAGTCAGAATACGCTGAAAGTTCATGATTTAAATGAAGATGCGGAATTTGATGcatatgaatatcctccttag
Del-c5038-F	TATCCCATGGCGCACCACAAAAGCGTCGAATTATCCCTAATCCCGGCCTGgtgtaggctggagctgcttcga
Del-c5038-R	GTAAACTGGCGTGGCGTGAAGGTATCCGTACCCATACACACCATATTTTGcatatgaatatcctccttag
Del-kgtP-F	CCAAAAAATAAACAAAAGCGACCGACAAAAGCATTGGATTACGGCAGGAGACATAATGGCgtgtaggctggagctgcttcga
Del-kgtP-R	TTTTGCCTGGGATATCGCCGGTGCAAGCACCGGCTATACCGTCTGGCAACTGACCCGTCAcatatgaatatcctccttag
rpoN-del-F	CTTCAGACTCTGATAGGGTAGAAGTTTGCGACGTTTTAGCAGGAGAGTACGATTCTGAACgtgtaggctggagctgcttcga
rpoN-del-R	GTGATCTCGACGTTATTTCCGGTAATGTTGAGCTGCATAGTGTCTTCCTTATCGGTTGGGcatatgaatatcctccttag
pGEN-rpoN-F EcoRI	ATGCGAATTCCTGCTCGACGAACCGTTTGC

pGEN-rpoN-R NdeI	AGTCCATATGTGAGCTGCATAGTGTCTTCC
pET21-rpoN-F	GCCATATGAAGCAAGGTTTGCAACTC
pET21-rpoN-R	ATCGGAATTCTCAGTGGTGGTGGTGGTGGTGAACGAGCTGTTTACGCTGGT
c5038-EMSA-F	ATCTGTGTGGTAAGAGAATC
c5038-EMSA-R	AACCACAGGCCGGGATTAGG
EMSA-kgtP-F	GGCGCGTCTTATACTCCCAC
EMSA-kgtP-R	CTCCTGCCGTAATCCAATGC
glnA-EMSA-F	ATGTTAAGCATGATAACGCC
glnA-EMSA-R	CAACAAACTTCACTTCGTGC
xylA-EMSA-F	TGCGCAATTGTACTTATTGC
xylA-EMSA-R	AGGCTTGCATATTGAACTCC
Pbla-EcoRI-F	GTAC*GAATTC*GCGAGATTCGTTACAGAGAC
c5038-Pbla-R	GCCATGGGATATTGATGACATAATAAGGGCGACACGGAAAT
Pbla-5038-F	ATTTCCGTGTCGCCCTTATTATGTCATCAATATCCCATGGC
Pbla-5038-R	GGAC*AAGCTT*TCACATTAGACCTAAAATTTTC
kgtP-Pbla-R	GTTACAGTACTTTCAGCCATAATAAGGGCGACACGGAAAT
Pbla-kgtP-F	ATTTCCGTGTCGCCCTTATTATGGCTGAAAGTACTGTAAC
Pbla-kgtP-R	GCAT*AAGCTT*CTAAAGCCGCATCCCTTTTC

Note: For deletion primers, uppercase letters indicate homologous regions and lowercase letters indicate sequence annealed to template plasmids pKD3 and pKD4.

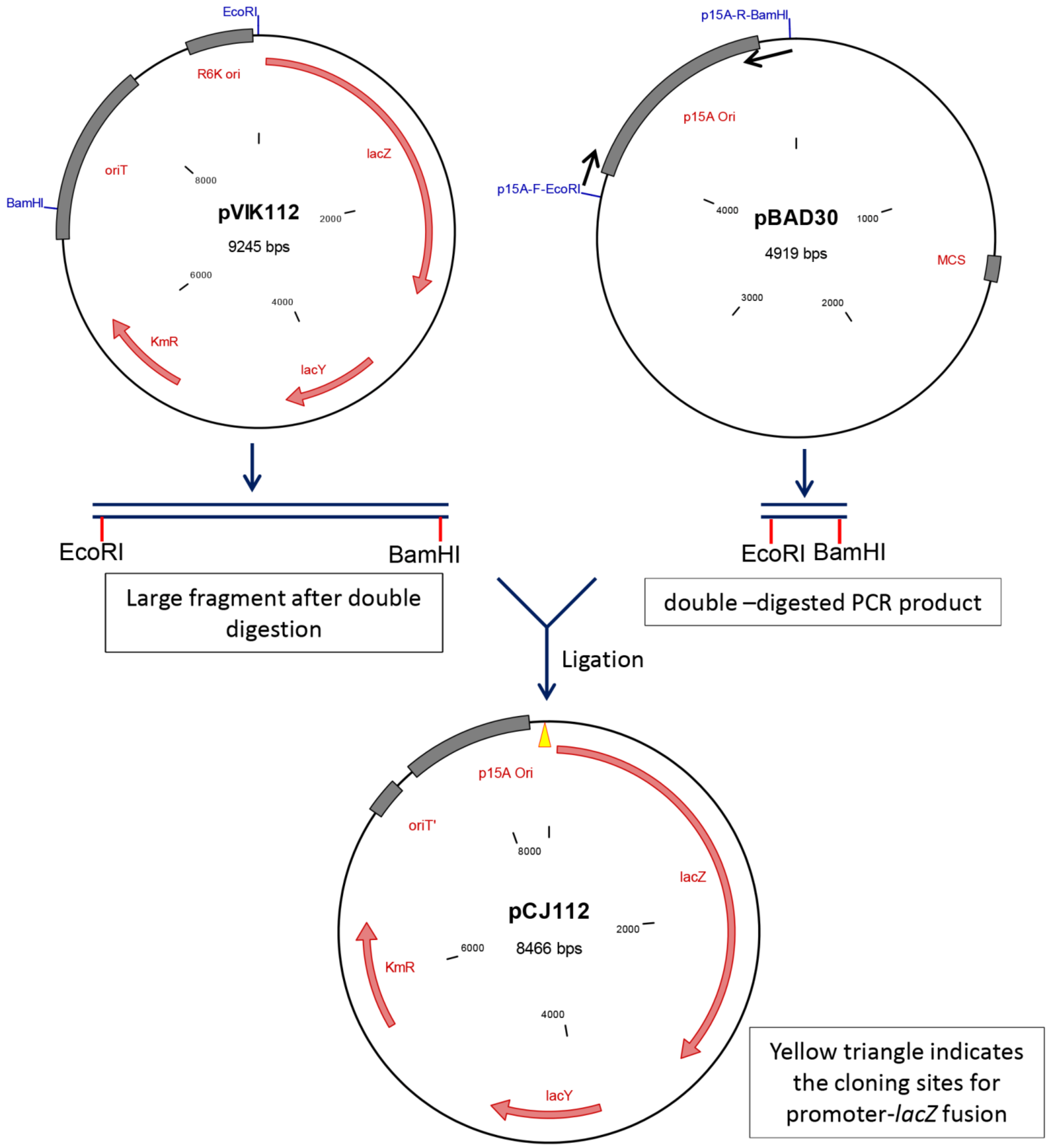

FIG S1. Construction of the pCJ112 plasmid vector.

A

		Helical hairpin (in)		Helical hairpin (out)
INDY (Vc)	144	LLSMWI-SNTATAA-MMLPL	369	VVFLTEFASNTASAALLIPV
SdcS (Sa)	158	FLSMFV-SNTAAVM-IMIPI	413	VLFLTEVTSNTATATMILPI
DccT (Cg)	171	FLSMWV-SNTATAV-VMLPI	421	VLFLTEFTSNTATAATFLPI
**C5038** (Ec)	157	VLSLVVPSATARTACV-VPI	397	IIIHLGFASATALTAALLPI
YbhI (Ec)	137	VLAPATPSNTARAGGIVLPI	375	IIVRYFFASGSAYIVAMLPV
CitT (Ec)	151	LLAPFTPSNTARTGGTVFPV	389	YFAHYLFASLSAHTATMLPV
TatT (Ec)	150	ILAPVTPSNSARGAGIIYPI	388	YLLRYFFASATAYTSALAPM

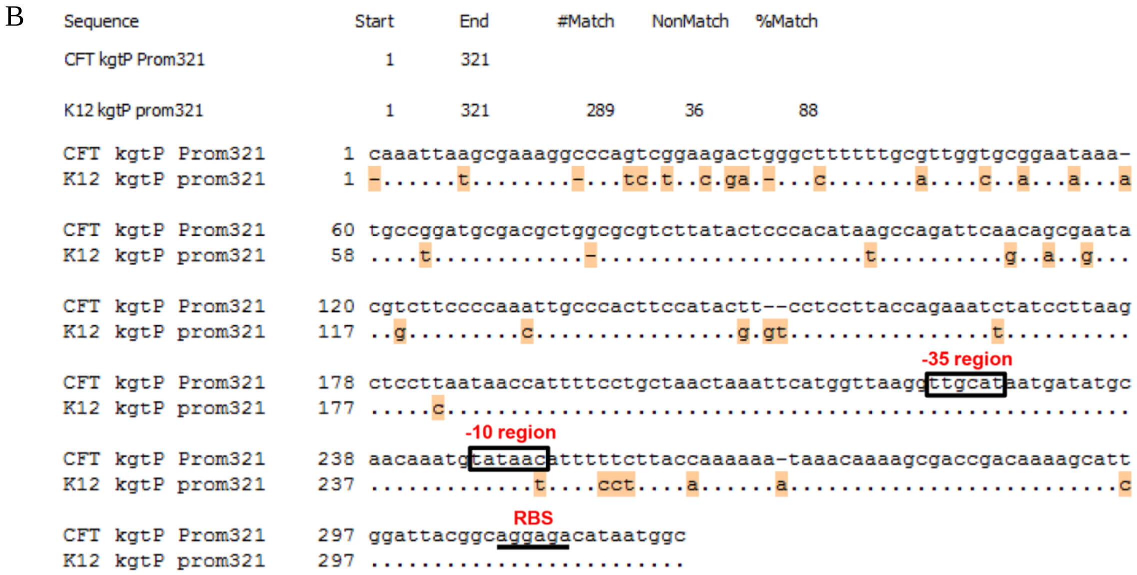

FIG S2. (A) A partial sequence alignment of C5038 and other characterized members in DASS family. ClustalW was used to create the graph. Helical hairpins (in) and (out) are transmembrane domains inserted into the membrane from the cytosolic and periplasmic side, respectively. SNT motifs involved in carboxylate-binding are highlighted. Bracketed letters indicate the organisms: Vc, *Vibrio cholerae*; Sa, *Staphylococcus aureus*; Cg, *Corynebacterium glutamicum*; Ec, *Escherichia coli*. (B) Sequence comparison of *kgtP* promoter regions (321 bp upstream of start codon) from K12 and CFT073. Dots, identical letters; dash, gaps; highlighted, differences.

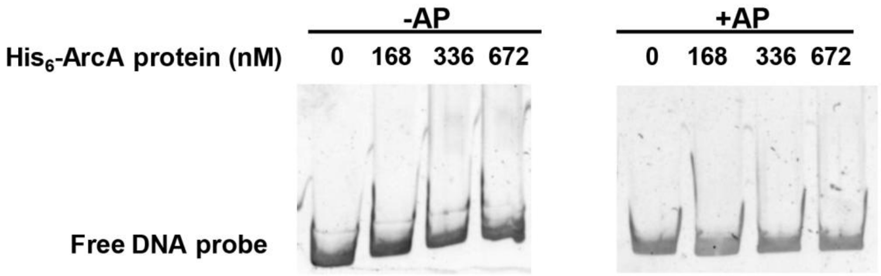

FIG S3. Non-radioactive EMSA studying the binding of ArcA to *c5038* promoter regions. Gel-extracted PCR products of *c5038* promoter region were used as probes. Purified His$_6$-ArcA fusion protein was added in different concentration in each reaction mixture as indicated. DNA fragments were stained with SYBR green. AP, acetyl phosphate.

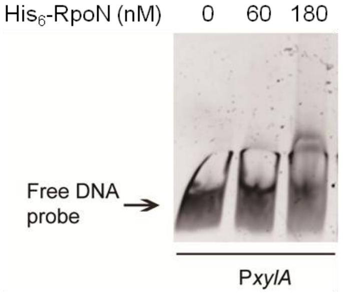

FIG S4. Non-radioactive EMSA studying the binding of RpoN to promoter regions of *xylA*. PCR products were used as probes. Purified His$_6$-RpoN fusion protein was added in different concentration in each reaction mixture as indicated. DNA fragments were stained with SYBR green.

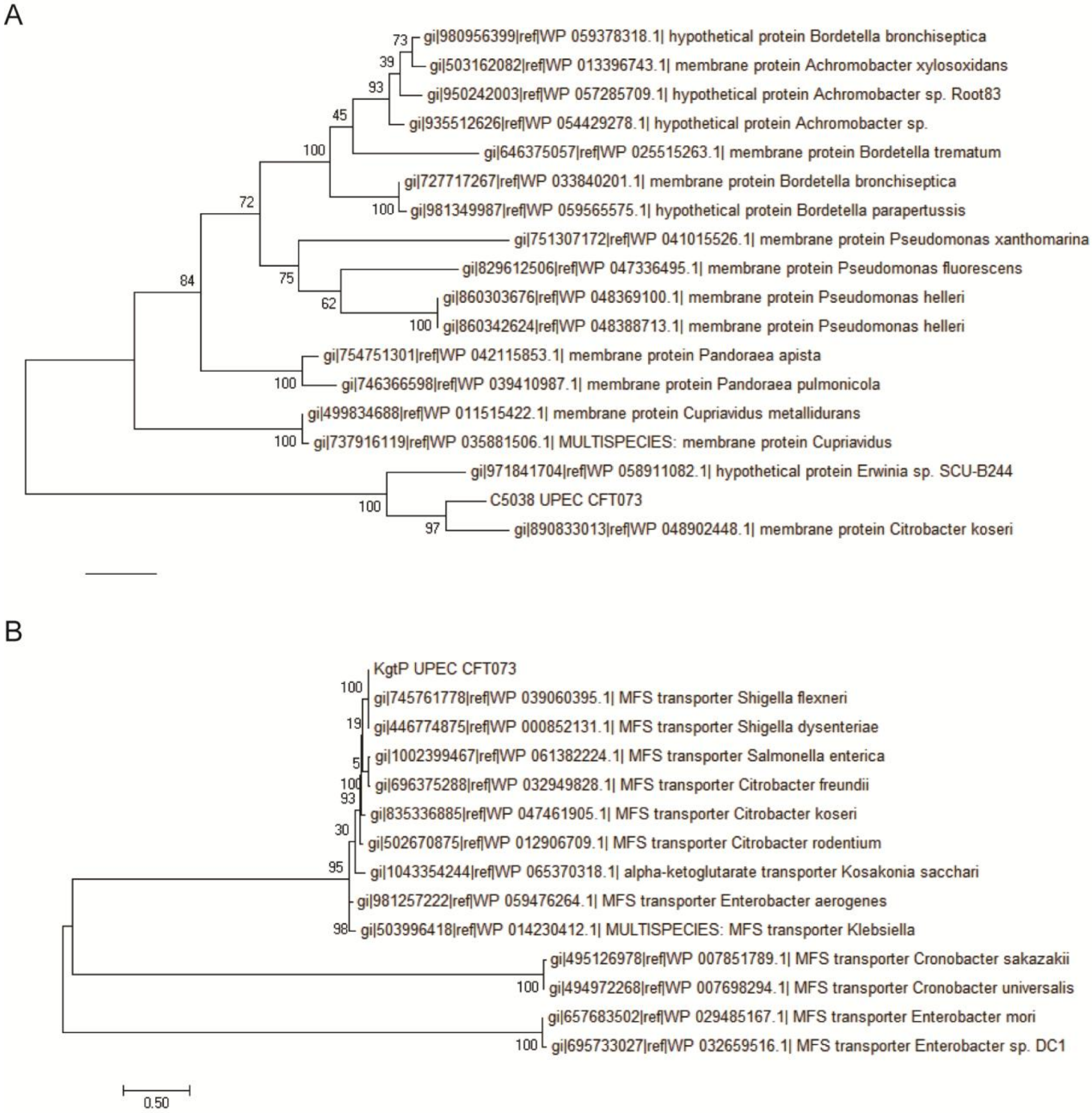

FIG S5. **Phylogenetic trees of C5038 (A) and KgtP (B)**. Phylogenetic trees were created based on the ClustalW alignments using Maximum Likelihood method (bootstrap n = 100) in MEGA7 program. *Escherichia* spp. strains were excluded.

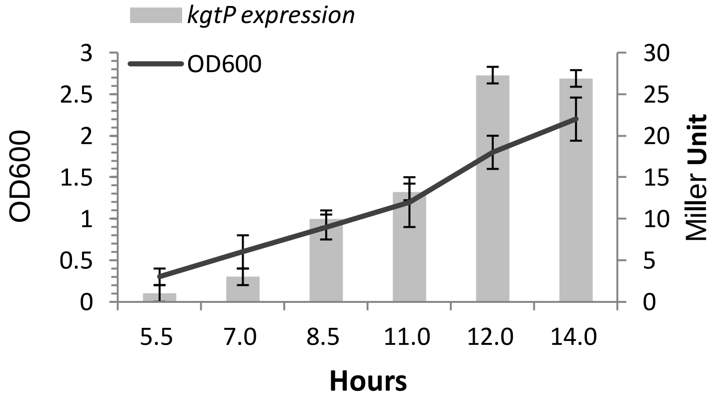

:

FIG S6. *kgtP* expression in relation to bacteria growth phase. Bacteria were grown aerobically in M9 medium containing glycerol as sole carbon source. Samples were taken at various time points for measuring optical density at

600nm and β-galactosidase activities.
